# Supplementary material for: High-dimensional super-resolution imaging reveals heterogeneity and dynamics of subcellular lipid membranes
Source: Nat Commun. 2020 Nov 18;11:5890. doi: 10.1038/s41467-020-19747-0 (PMC7674432; doi:10.1038/s41467-020-19747-0)
Supplement: Supplementary file 1 — Supplementary Information [file 41467_2020_19747_MOESM1_ESM.docx]

Supplementary Information

**High-dimensional Super-Resolution Imaging Reveals Heterogeneous Subcellular Lipid Membranes**

**Table of Content**

Supplementary Fig. 1 – Principle of Spectrum and Polarization Optical Tomography (SPOT) 1

Supplementary Fig. 2 – The improved optical sectioning of SPOT and its enhanced measurement accuracies in both emission ratio and polarization modulation depth 2

Supplementary Fig. 3 **–** Comparison of the measurement accuracies in the emission ratio and modulation depth among WF, SIM3D, SPOT, and SPOT-SIM3D 4

Supplementary Fig. 4 – Comparison of the measurement accuracy in the dipole orientation under polarized out-of-focus background between WF and SPOT 5

Supplementary Fig. 5 **–** Lipids composition in different organelles 6

Supplementary Fig. 6 **–** The heterogeneous lipid distribution of lipid droplets 7

Supplementary Fig. 7 **–** Calibration of the Nile Red emission ratio in solvents with known polarity 8

Supplementary Fig. 8 **–** Influence of noise on measurement accuracies 9

Supplementary Fig. 9 **–** Influence of the HiLo weighting factor on measurement results 10

Supplementary Movie – Time-lapse high-dimensional super-resolution imaging of the late-stage division of two U2-OS cells 11

Supplementary Note – Detailed reconstruction process of SPOT12

References17


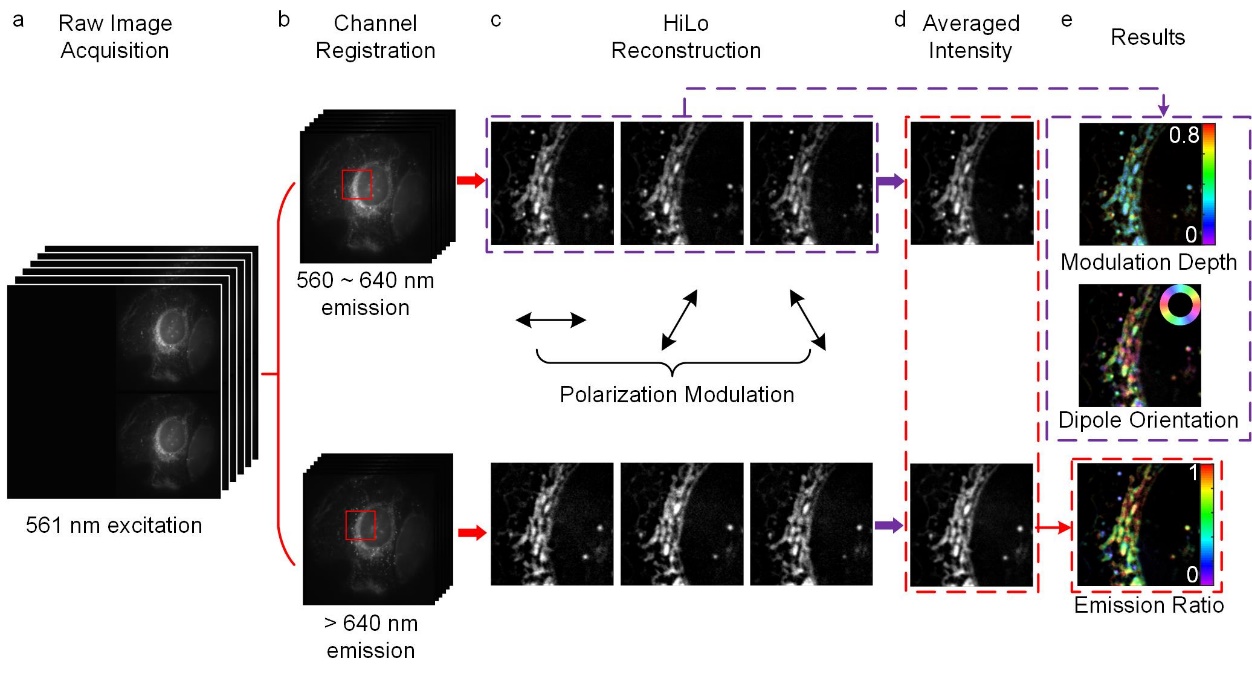


**Supplementary Fig. 1 – Principle of Spectrum and Polarization Optical Tomography (SPOT). (a)** Six raw images are first acquired under the six different patterns of structured illumination, including three pattern directions of 0°, 60°, 120° and two phases with a π shift for each direction. **(b)** Each raw image has two channels with different emission bands for the emission ratio analysis. The two channels are registered based on the bead alignment experiment to guarantee the accurate calculation of the emission ratio. Though hyperspectral imaging with up-to-four channels is compatible with SPOT, two-color channels were found sufficient in extracting and differentiating the membranes’ polarity information. The remaining channels are reserved for other purposes, such as GFP colocalization. **(c)** In each channel, the six images are grouped according to the three illumination pattern directions, and each group contains a pair of images with a π phase shift to obtain the optically sectioned image using the algorithm High-Low (HiLo) Fourier filtering^1^. The obtained three optical sectioned images in each channel are also excited by different polarizations because of the polarization modulation in the SLM-SIM system^2^. **(d)** The SPOT intensity image of each detection channel is obtained by averaging the three optical sectioned images. **(e)** The polarization modulation depth and the dipole orientation are obtained from three optical sectioned images under polarization modulation^2^ in the 560-640 nm channel. The emission ratio is calculated by dividing the SPOT intensity image of >640 nm detection channel by that of 560-640 nm channel. The detailed reconstruction process of SPOT and SPOT-SIM3D is included in Supplementary Note.

**
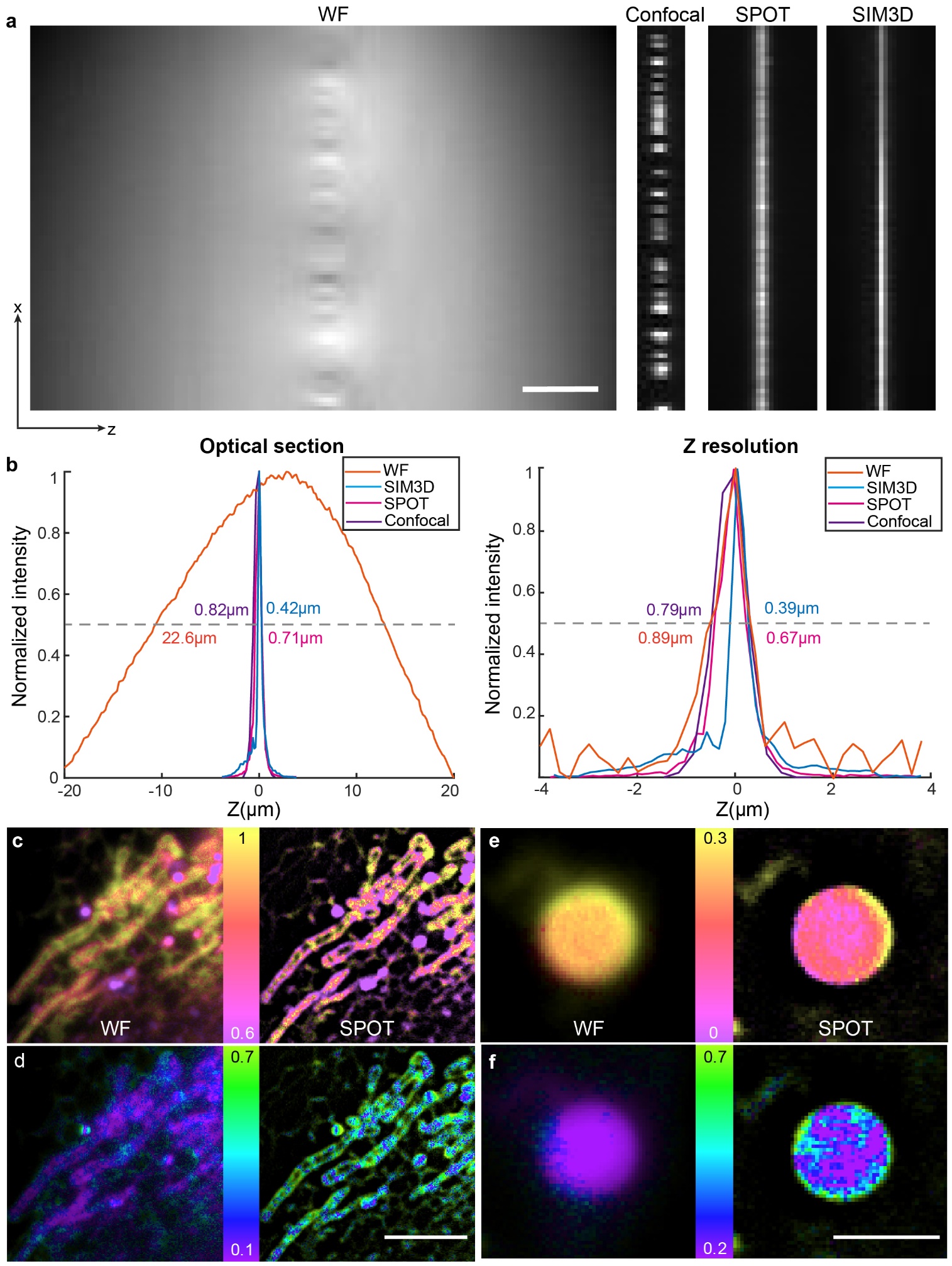
**

**Supplementary Fig. 2 –** **The improved optical sectioning of SPOT and its enhanced measurement accuracies in both emission ratio and polarization modulation depth.** **(a)** The comparison among wide-field (WF), confocal, SPOT, and SIM on the XZ images of 100-nm fluorescent beads. The SPOT result shows a significant attenuation of the out-of-focus signal that is comparable to confocal result acquired in a commercial system (Leica TCS SP8, pinhole: 1 a.u.). The similar results were repeated 3 times independently. **(b)** The normalized intensity profiles along the z-axis in **(a)** quantify the power of optical sectioning, and the z intensity profile of a single bead quantifies the longitudinal resolution of different imaging modalities. **(c-d)** the comparison results of the lipid polarity map and the lipid phase map of mitochondria between WF (left) and SPOT (right) modalities. **(e-f)** the comparison results of the lipid polarity map and the lipid phase map of lipid droplets. The similar results in (c)-(f) were repeated 3 times independently. Scale bar: (a) 5 μm; (c-d) 5 μm; (e-f) 2 μm.

**
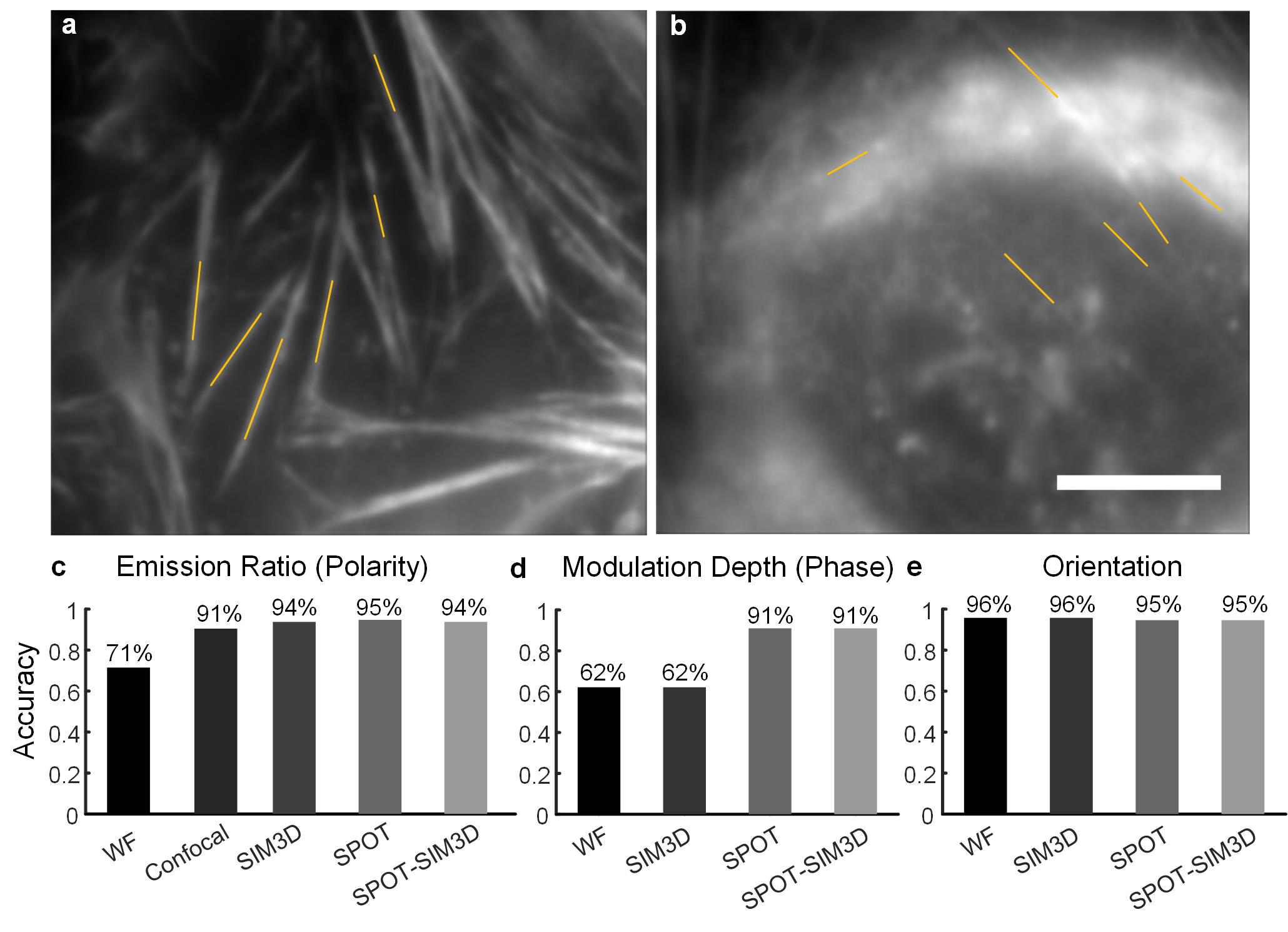
**

**Supplementary Fig. 3 – Comparison of the measurement accuracies in the emission ratio and modulation depth among WF, Confocal, SIM3D, SPOT, and SPOT-SIM3D. (a)** The WF image of U2-OS cells with actin filaments labeled by Phalloidin-AF568. We measured the emission ratio, modulation depth, and dipole orientation of the AF568 fluorophores in areas free of out-of-focus signals (marked by the orange lines) and taken them as ground-truth values. **(b)** The WF image of U2-OS cells with the actin filaments labeled by Phalloidin-AF568 and the lipid membranes stained by Nile Red. The measurement of the optical properties of the AF568 fluorophores is strongly disturbed by the out-of-focus signals of Nile Red that stains across the crowded subcellular compartments. **(c-e)** The histograms compare the emission ratio **(c)**, modulation depth **(d)**, and dipole orientation **(e)** among WF, SIM3D, SPOT, and SPOT-SIM3D. SIM3D, SPOT, and SPOT-SIM3D achieved significantly higher accuracies than WF when measuring the emission ratio of AF568 fluorophores in Nile Red stained cells. SPOT and SPOT-SIM3D achieved significantly higher measurement accuracies in the modulation depth. Because the out-of-focus signals generated by the dipoles of Nile Red are polarization isotropic, all the imaging methods achieve high measurement accuracy in the dipole orientation. To further compare SPOT with confocal microscopy, we measured the emission ratio on a commercial confocal microscope (Leica TCS SP8, pinhole: 1 a.u.). The measurement accuracy of confocal in emission ratio **(c)** is also improved compared with WF but slightly lower than SPOT. However, we cannot conclude that SPOT is better than confocal because the measurements of emission ratios were performed on two microscopes that may introduce other influencing factors. Also, the polarization measurement accuracy of confocal is not included here because polarization imaging is not available on commercial confocal microscopes. The similar results in (a) and (b) were repeated 3 times independently. Scale bar: 5 μm.

**
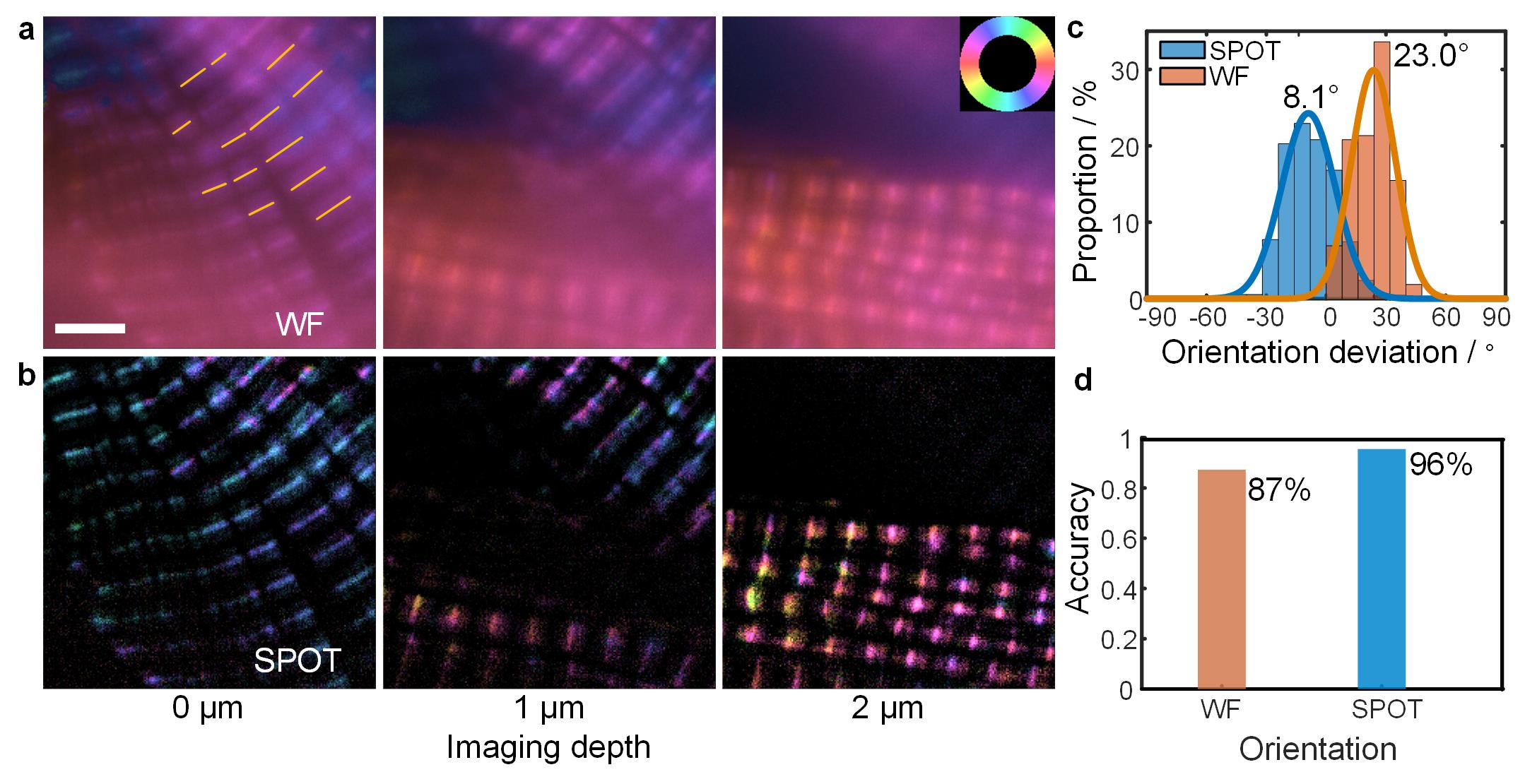
**

**Supplementary Fig. 4 – Comparison of the measurement accuracy in the dipole orientation under polarized out-of-focus background between WF and SPOT.** The dense and well-organized actin filaments are labeled with Phalloidin-AF568 in cardiac muscle cells. **(a, b)** The WF and SPOT dipole orientation images of two overlapping cells at the imaging depths of 0 μm, 1 μm, and 2 μm. The dipole orientation information is pseudocolor coded following the pattern of the color wheel, e.g., the red color represents horizontal orientation, and the cyan color indicates vertical orientation. The dipole orientation of AF568 fluorophores is perpendicular to the Z lines in the cardiac muscle cells, as being marked by the lines in **(a)**. The statistical data **(c)** shows the orientation deviation measured by WF and SPOT, and their measurement accuracies in dipole orientation are shown in **(d)**. The dipole orientations measured by WF are heavily biased by the defocused dipoles, while SPOT can significantly alleviate this issue. The similar results in (a) and (b) were repeated 3 times independently. Scale bar: 3 μm.


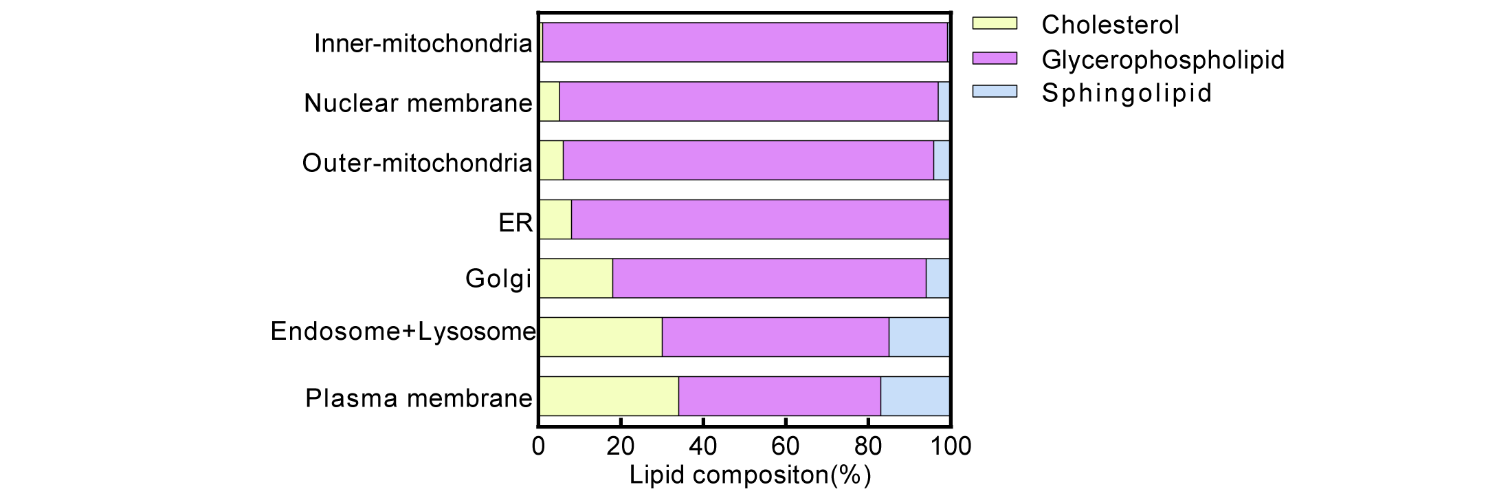


**Supplementary Fig. 5 – Lipids composition in different organelles.** Distribution of three lipid categories (glycerophospholipid, sphingolipid, and cholesterol) in the membrane of mitochondria, nuclear membrane, ER^3^, Golgi, endosome, lysosome, and plasma membrane^4^.


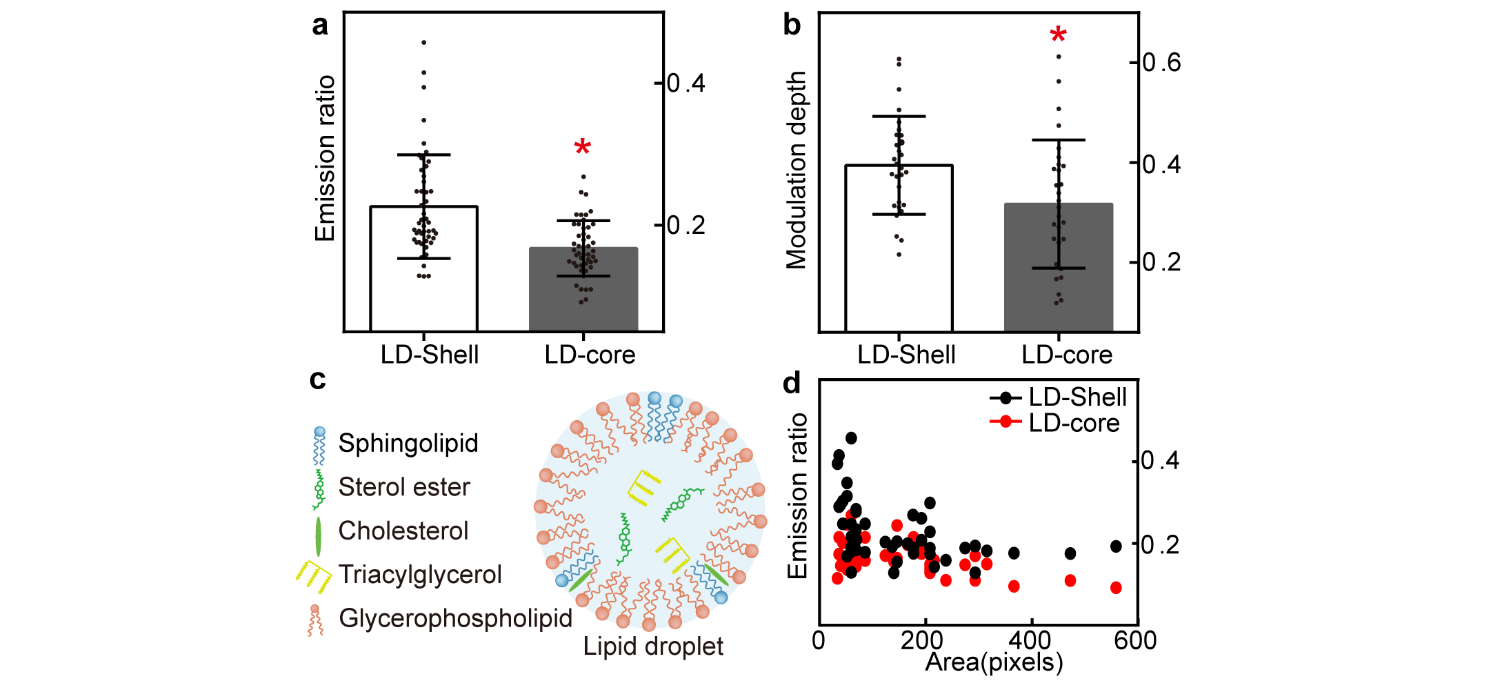


**Supplementary Fig. 6 – The heterogeneous lipid distribution of lipid droplets. (a)** Statistical results (n=48 organelles) show a higher lipid polarity in the shell of lipid droplets than that in the core. This observation is consistent with the fact that the triacylglycerol and sterol ester stored in the lipid core has a much lower polarity^5^. **(b)** Despite a lower lipid polarity, the lipid core also has a significantly lower phase than the shell because the lipid in the core is stored in a disordered manner (n=29 organelles). Two-sided t-test is adopted and p<0.0001 in (a), p= 0.0122 in (b). The red ‘*’ in (a) and (b) indicate that there exist significant differences. Data in (a) and (b) are presented as mean values +/- SD. **(c)** The schematic diagram shows the heterogeneous lipid composition in the core and shell of lipid droplets. **(d)** Statistical results (n=48 organelles) show that the emission ratios drop lower as lipid droplets grow larger.


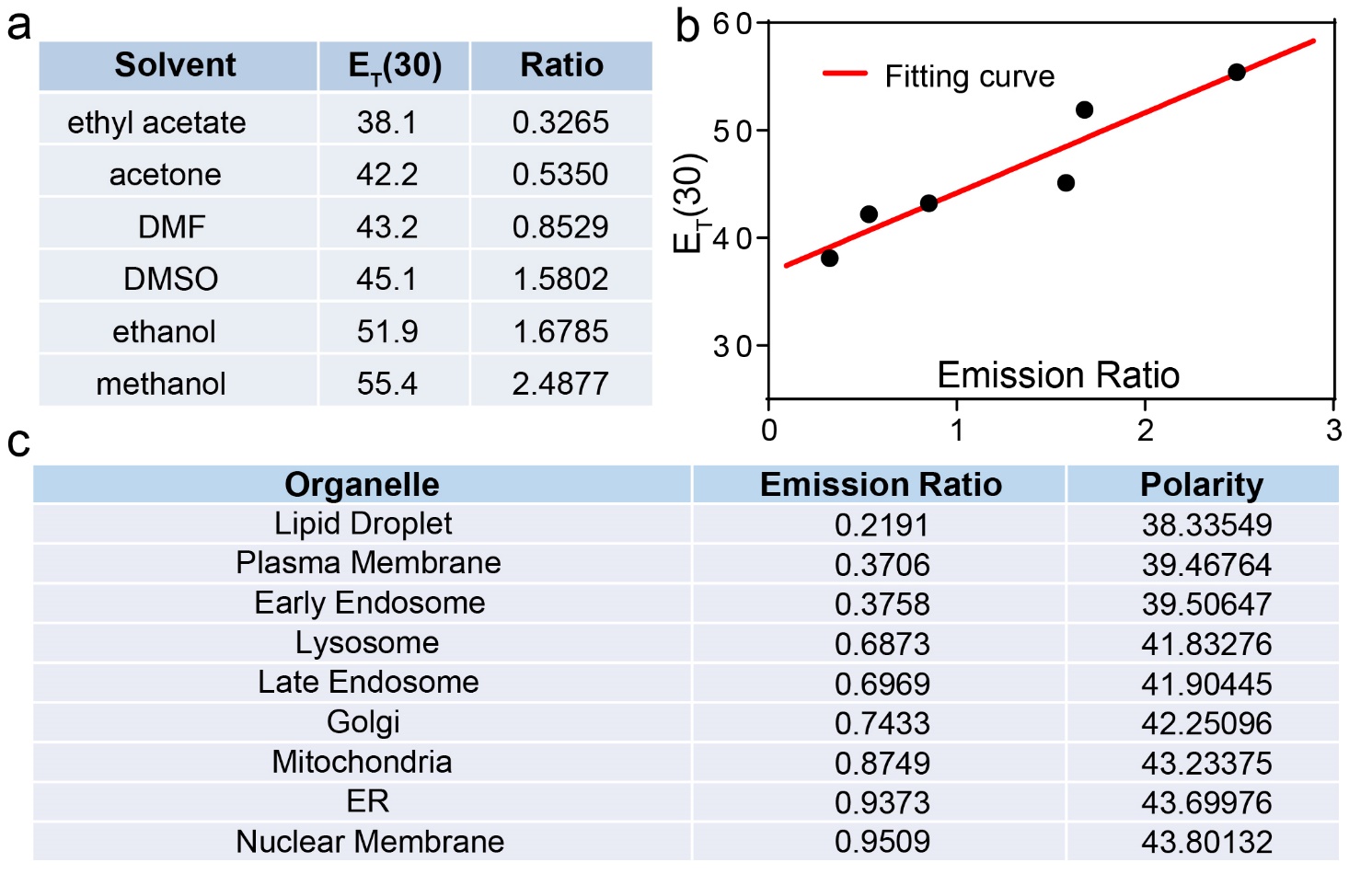


**Supplementary Fig. 7 – Calibration of the Nile Red emission ratio in solvents with known polarity.** We dissolve Nile Red in solvents, including ethyl acetate, acetone, DMF, DMSO, ethanol, and methanol, whose polarities of solvents are known and indicated by the molar transition energy E_T_(30). The emission ratios in solvents are measured and fitted as in **(a, b)**. **(b)**The average polarities of different compartmental membranes are further calculated based on the fitted curve and the measured emission ratio. The E_T_(30) polarity of the lipid droplet is 38.335 that is similar to that in the previous publication^6^.


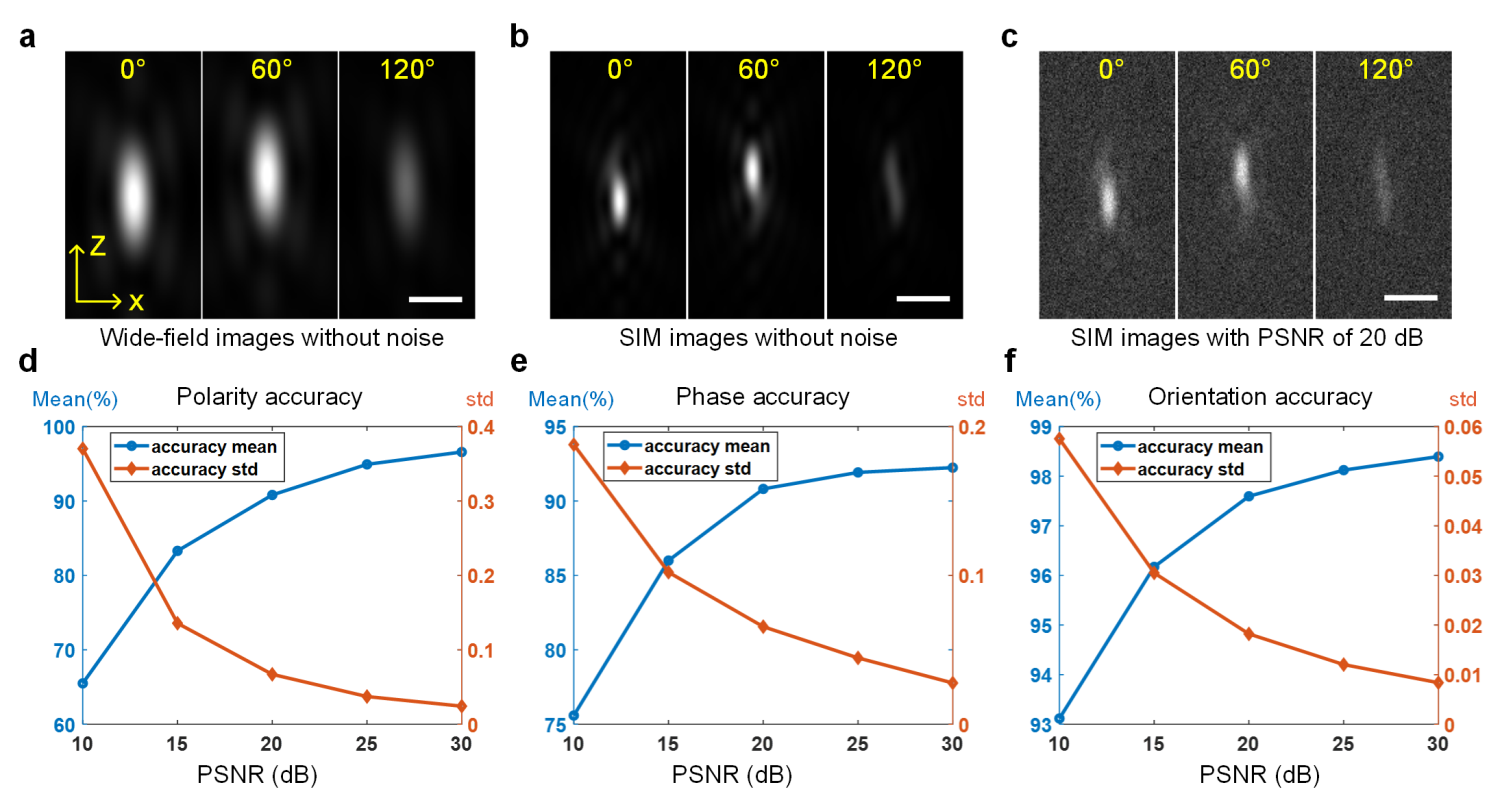


**Supplementary Fig. 8 – Influence of noise on measurement accuracies. (a, b)** The WF and SR images under the excitation polarizations of 0°, 60°, and 120°. **(c)** The images correspond to (b) with a PSNR of 20 dB. For each PSNR level, we repeat 500 trials of simulation and study the influence of noise on measurement accuracies with two statistical indicators: the mean accuracy and the standard deviation (std) value of the accuracy. The std of accuracy means how robust under noise the measurements are, which can also be termed as ‘precision’. **(d)** The relationships between polarity accuracy and PSNR in SR measurements. Each data point is the statistical result of 500 simulations (sample size n = 500). The blue line represents the relationship between the mean accuracy and PSNR, while the orange line represents the relationship between the precision and PSNR. **(e, f)** The changes of phase accuracy and orientation accuracy with PSNR, respectively. The measurement accuracies of noise-free WF images are 85%, 55%, and 81% for polarity, phase, and orientation. When the PSNR is greater than 17 dB, all the accuracies of SR measurements are better than those of wide-field, which are 87%, 88%, and 97%. In this work, we empirically require a PSNR of >20 dB for the raw image acquired throughout all imaging experiments. Scale bar: (a-c) 0.5$\mu m$.


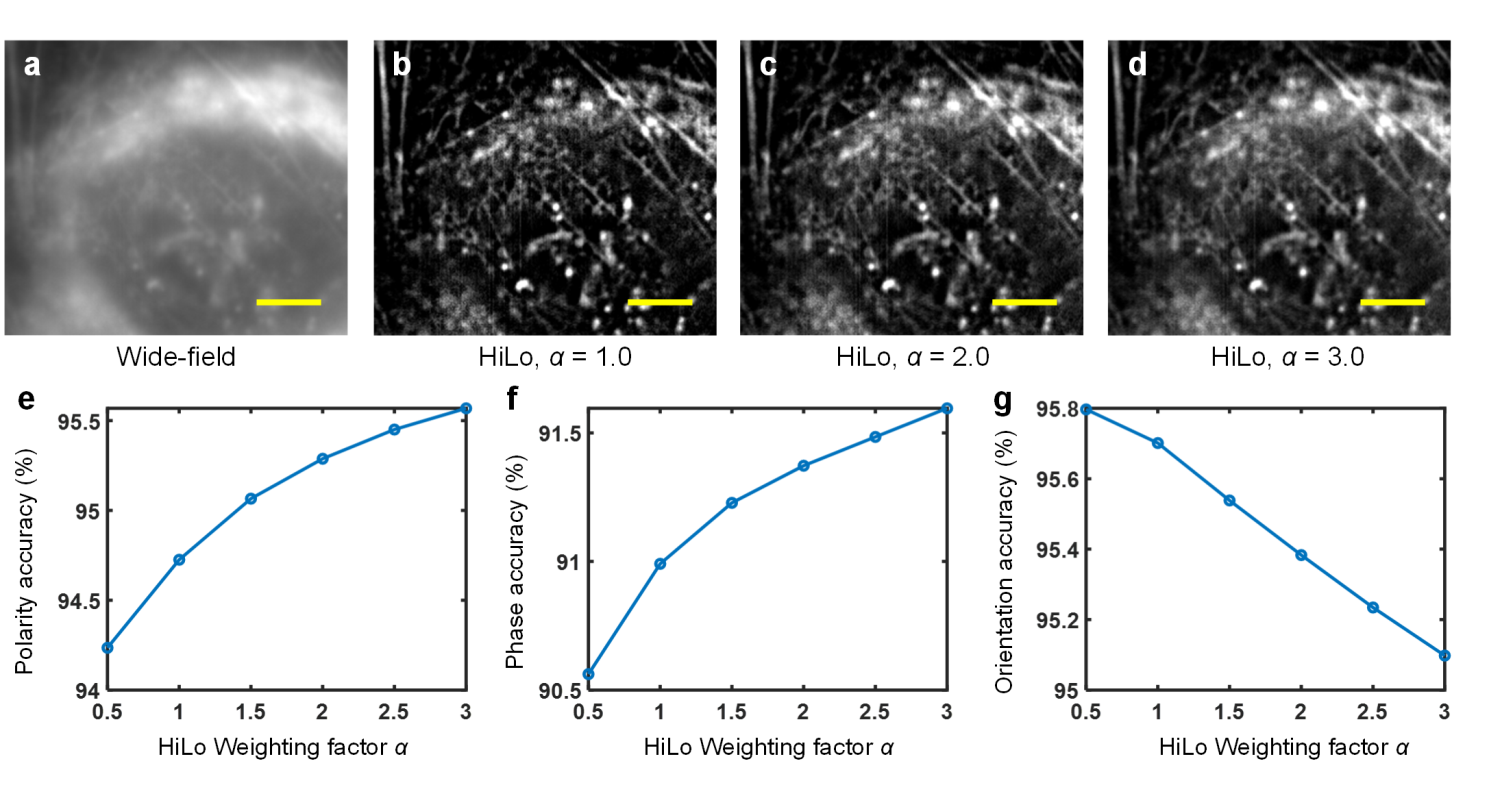


**Supplementary Fig. 9 – Influence of the HiLo weighting factor on measurement results. (a)** The WF image. **(b-d)** The HiLo images with the HiLo weighting factors (α) of 1.0, 2.0, and 3.0, respectively. **(e-f)** The relationship of polarity, phase, and orientation accuracy with the HiLo weighing factor α. The measurement accuracies in the emission ratio, polarization modulation depth, and dipole orientation are little changed when the HiLo weighting factors (α) varies from 0.5 to 3. We set the HiLo weighting factors (α) to 1 throughout all imaging experiments. Scale bar: (a-d) 5$\mu m$.

**Supplementary Movie – Time-lapse high-dimensional super-resolution imaging of the late-stage division of two U2-OS cells.** The intensity image, the lipid polarity map, and the lipid phase map are shown from left to right. The colormap in the right-bottom corner indicates the corresponding value for each pseudocolor. The imaging speed is 1/3 Hz, and a selected period of 255 s is recorded and shown here.

**Supplementary Note – Detailed reconstruction process of SPOT**

For either SPOT or SPOT-SIM3D dataset, three groups of raw images are acquired with three excitation polarization $\theta_{i} (i=1, 2, 3)$. Each group contains two images with a phase shift of $\pi$ for SPOT or five images with a phase shift of $2\pi/5$ for SPOT-SIM3D. Each image contains four subareas corresponding to the blue/green/yellow/red channels. Only 561 nm excitation is used for imaging Nile Red stained specimen, and an additional 488 nm excitation is used for GFP colocalization.

The SPOT process consists of the following steps:

1. Registration of different detection channel:
   Each raw image contains four spectral detection channels, which are located in non-overlapping spatial positions. Different spectral channels are cropped and registered by multiplying their corresponding affine transformation matrix. The measurement method of the affine transformation matrix is shown in step s1). For SPOT imaging of Nile Red signal, only the yellow channel or red channels are reserved for further analysis. For GFP colocalization, only the green channel is reserved.
2. Calibration of the illumination nonuniformity:
   The illumination intensity corresponding to different polarization modulation may be different. By dividing the image corresponding to each polarization modulation by the correction matrix, we can get the images after illumination correction. The measurement method of the correction matrix is shown in step s2). For each detection channel, the images after illumination correction are expressed as:$I_{SIj,Pi}$, where the subscript $Pi (i=1, 2, 3)$ represents different polarization modulation, and the subscript $SIj$ ($j=1, 2$ for SPOT and $j=1,\ldots, 5$ for SPOT-SIM3D) represent multiple sinusoidal illuminations with different phases.
3. Obtain optical sectioning images

By averaging the structured illumination images in one group, we can get the image $I_{U,Pi}$ of uniform illumination under this polarization modulation:

$$I_{U,Pi}=\frac{1}{n}\sum_{j=1}^{n} I_{SIj,Pi}$$

, where *n* represents the number of images in one group and $n=2$ for SPOT, $n=5$ for SPOT-SIM3D.

Combining $I_{U,Pi}$ and $I_{SI1,Pi}$, we can get the optical sectioning image corresponding to the polarization modulation by using HiLo reconstruction algorithm. The wide-field image contains the in-focus signal $I_{\mathrm{in}}(x)$ and the out of focus signal $I_{\mathrm{out}}(x)$ of the sample, where $x$ represents the spatial coordinates, and they can be expressed as $I_{in,Pi}(x)$ and $I_{out,Pi}(x)$ under the polarization modulation. In this way, the images acquired with uniform and structured illumination are written respectively as:

$$I_{U,Pi}(x)=I_{in,Pi}\left( x \right)+I_{out,Pi}(x)$$

$$I_{SI1,Pi}(x)=\frac{1}{2}[I_{in,Pi}\left( x \right)(1+M\sin\left( k_{g}x \right))+I_{out,Pi}(x)]$$

The structured illumination pattern is modeled with a sinusoid of spatial frequency $k_{g}$ and contrast *M*. The in-focus signal is modulated by the sinusoid pattern, whereas the out-of-focus is not. Then a partially demodulated image can be obtained from:

$$D_{Pi}(x)=\left| I_{U,Pi}\left( x \right)-2I_{SI1,Pi}\left( x \right) \right|=I_{in,Pi}\left( x \right)M\left| \sin\left( k_{g}x \right) \right|$$

By applying a low-pass filter (LP) to $D_{Pi}(x)$ of cutoff frequency somewhat smaller than $k_{g}$, we can find:

$$\mathrm{LP}[I_{in,Pi}\left( x \right)]\approx\frac{\pi}{2M}\mathrm{LP}[D_{Pi}(x)]$$

The high-frequency components in $I_{in,Pi}\left( x \right)$ can be obtained by applying a complementary high-pass filter (HP) to $I_{U,Pi}$:

$$HP[I_{in,Pi}\left( x \right)]\approx\mathrm{HP}[I_{U,Pi}(x)]$$

Then the full bandwidth representation of $I_{in,Pi}\left( x \right)=\mathrm{LP}\left[ I_{in,Pi}\left( x \right) \right]+HP[I_{in,Pi}\left( x \right)]$ can be obtained from:

$$I_{OS,Pi}\left( x \right)=\alpha\mathrm{LP}\left[ D_{Pi}\left( x \right) \right]+HP[I_{U,Pi}\left( x \right)]$$

The α was set to 1.0 in our experiment

The wide-field sectioning image $I_{os\_k}$ can be obtained by averaging the sectioning images under different polarization modulation, where $k=1, \ldots, m$ represents different detection channels:

$$I_{OS\_k}=(I_{OS,P1}+I_{OS,P2}+I_{OS,P3})/3$$

1. (Only for SPOT-SIM3D) Reconstruct SIM3D super-resolution image using standard SIM reconstruction software. In our work, the 3D SIM reconstruction is performed on the commercial software of GE OMX SR.
2. Obtain the image of emission ratio

For SPOT, repeat steps c) ~ e) for each detection channel to obtain the corresponding wide field sectioning images of all the detection channels. Then the emission ratio can be obtained by:

$$R=I_{OS\_4}/I_{OS\_3}$$

Here $I_{OS\_3}$ represents the yellow detection channel and $I_{OS\_4}$ represents the red detection channel.

For SIM3D, the emission ratio $R=I_{SR\_4}/I_{SR\_3}$, where $I_{SR\_3}$ and $I_{SR\_4}$ represents the super-resolution images from the yellow detection channel and red detection channel, respectively.

1. Calculate polarization information (modulation depth, dipole orientation):
   For both SPOT and SPOT-SIM3D. The polarization information includes modulation depth $OUF$ and dipole orientation *β*. When the polarization modulation is $\theta_{i}$, the fluorescence dipole excitation intensity $I_{OS,Pi}$ is:

$$I_{OS,Pi}=I_{DC}+I_{AC}\cdot\cos\left( 2\theta_{i}-2\beta\right), i=1, 2, 3$$

, or in the matrix form:

$$\left[ \begin{aligned} I_{OS,P1} \\ I_{OS,P2} \\ I_{OS,P3} \end{aligned} \right]=\left[ \begin{aligned} 1 \\ 1 \\ 1 \end{aligned} \begin{aligned} cos2\theta_{1} \\ cos2\theta_{2} \\ cos2\theta_{3} \end{aligned} \begin{aligned} sin2\theta_{1} \\ sin2\theta_{2} \\ sin2\theta_{3} \end{aligned} \right]\left[ \begin{aligned} I_{DC} \\ I_{AC}\cdot cos2\beta\\ I_{AC}\cdot sin2\beta\end{aligned} \right]$$

, where $I_{DC}$ is the DC component, $I_{AC}$ is the AC component, and $\theta_{i}$ is the direction of polarization. Then the $\beta$, $I_{DC}$ and $I_{AC}$ can be obtained by matrix inversion. The modulation depth can be obtained by $OUF=2I_{\mathrm{AC}}/(I_{\mathrm{AC}}+I_{\mathrm{DC}})$.

1. Validation of reconstruction results

We also take the following steps to further check the processed data to improve the reliability of the measurement:

1. Double-check the channel registration. Arrange different colors for the registered images of different detection channels and get a composite image. Observe the nuclear membrane part of the composite image. If the nuclear membranes of different channels are misaligned, go back to step a) and remeasure the affine transformation matrix.
2. Check the fluctuation of modulation depth. This step is mainly used to exclude the data that motion blur or photobleaching have a significant impact on the measurement. As shown in step f ), at least 3 polarization modulations are required to get modulation depth (OUF*)* and dipole orientation *β*, and we adopted $\theta_{i}=0^{\circ},60^{\circ},120^{\circ}$ in the experiment. For the data check, we carry out 5 polarization modulations in sequence with $\theta_{i}=0^{\circ},60^{\circ},120^{\circ},0^{\circ},60^{\circ}$. When selecting $\theta_{i}=0^{\circ},60^{\circ},120^{\circ}$, we can find the OUF. Similarly, when choosing $\theta_{i}=60^{\circ},120^{\circ},0^{\circ}$ and $\theta_{i}=120^{\circ},0^{\circ},60^{\circ}$, we can also solve the modulation depth, expressed as OUF*’* and OUF*’’*. OUF, OUF*’* and OUF*’’* should be close to each other if the motion blur and photobleaching are not obvious. We excluded the data where OUF, OUF*’* and OUF’*’* differ more than 15%.

Two calibration experiments are required, as shown below:

s1) Measurement of the affine transformation matrix for each detection channel. Prepare a slide of 100 nm fluorescent beads, where the beads are distributed in a single layer and sparse enough. Get the 6 (for SPOT) or 15 (for SPOT-SIM3D) raw images. Average all the raw images to get an image under the uniform illumination, denoted as $I_{U}$. Crop and separate the images of different detection channels in $I_{U}$, denoted as $I_{U,k}$ ($k=1,2,\ldots m$ represents different detection channel). Localize the beads in $I_{U,k}$ and find out the corresponding beads in different channels. Pick *q* (*q*≥3) such beads, and their coordinate in the *k*-th channel can be expressed as:$\left[ \begin{aligned} \boldsymbol{X}_{k} \\ \boldsymbol{Y}_{k} \\ \boldsymbol{1} \end{aligned} \right]$, where $\boldsymbol{X}_{k}$ is a row vector composed of the horizontal ordinates of *q* beads, $\boldsymbol{Y}_{k}$ is a row vector composed of the vertical ordinates of *q* beads, and $\boldsymbol{1}$ is a row vector consisting of *q* ones. Then the affine transformation matrix $H_{k}$ of the *k*-th detection channel can be calculated by:

$$H_{k}=\left[ \begin{aligned} \boldsymbol{X}_{1} \\ \boldsymbol{Y}_{1} \\ \boldsymbol{1} \end{aligned} \right]\left[ \begin{aligned} \boldsymbol{X}_{k} \\ \boldsymbol{Y}_{k} \\ \boldsymbol{1} \end{aligned} \right]^{-1}$$

s2) Measurement of illumination uniformity correction matrix for each polarization modulation. Prepare a dense single-layer slide of 100 nm fluorescent beads. Get 3 groups of raw images with three excitation polarization $\theta_{i} (i=1, 2, 3)$. By averaging 2 (SPOT) or 5 (SPOT-SIM3D) images in each group, we can get wide field images $\overline{I}_{U,Pi}$ without illumination uniformity correction, where subscript $Pi (i=1, \ldots, 3)$ represents different polarization modulation. Then the uniformity correction matrix $U_{i}$ corresponding to the *i*-th polarization modulation is:

$$U_{i}(x)=\frac{\overline{I}_{U,Pi}\left( x \right)}{\overline{I}_{U,P1}\left( x \right)}$$

, where $x$ represents the spatial coordinates of localized fluorescent beads.

**References:**

1. Mertz, J. & Kim, J. Scanning light-sheet microscopy in the whole mouse brain with HiLo background rejection. Journal of Biomedical Optics **15**, 016027 (2010).
2. Zhanghao, K. et al. Super-resolution imaging of fluorescent dipoles via polarized structured illumination microscopy. Nat Commun **10**, 4694 (2019).
3. Thomas, E.A.M.D., Joseph, F.H.P.D., Darrell, D.F.M.D. & Stanley, G.S.M.D. Membrane Physiology. 105, 412 (1980).
4. Casares, D., Escribá, P. V. & Rosselló, C. A. Membrane Lipid Composition: Effect on Membrane and Organelle Structure, Function and Compartmentalization and Therapeutic Avenues. International Journal of Molecular Sciences **20**, 2167 (2019).
5. Olzmann, J. A. & Carvalho, P. Dynamics and functions of lipid droplets. Nat Rev Mol Cell Biol 20, 137-155 (2019).
6. Collot, M. et al. Probing Polarity and Heterogeneity of Lipid Droplets in Live Cells Using a Push-Pull Fluorophore. Anal Chem **91**, 1928-1935 (2019).
