# Supplementary material for: High-dimensional super-resolution imaging reveals heterogeneity and dynamics of subcellular lipid membranes
Source: Nat Commun. 2020 Nov 18;11:5890. doi: 10.1038/s41467-020-19747-0 (PMC7674432; doi:10.1038/s41467-020-19747-0)
Supplement: Supplementary file 3 — Reporting Summary [file 41467_2020_19747_MOESM3_ESM.pdf]

nature research

Corresponding author(s): Peng Xi
Last updated by author(s): Oct 14, 2020

Reporting Summary

Nature Research wishes to improve the reproducibility of the work that we publish. This form provides structure for consistency and transparency in reporting. For further information on Nature Research policies, see our Editorial Policies and the Editorial Policy Checklist.

Statistics

For all statistical analyses, confirm that the following items are present in the figure legend, table legend, main text, or Methods section.

n/a	Confirmed	
☐	☒	The exact sample size (*n*) for each experimental group/condition, given as a discrete number and unit of measurement
☐	☒	A statement on whether measurements were taken from distinct samples or whether the same sample was measured repeatedly
☐	☒	The statistical test(s) used AND whether they are one- or two-sided<br>*Only common tests should be described solely by name; describe more complex techniques in the Methods section.*
☒	☐	A description of all covariates tested
☐	☒	A description of any assumptions or corrections, such as tests of normality and adjustment for multiple comparisons
☐	☒	A full description of the statistical parameters including central tendency (e.g. means) or other basic estimates (e.g. regression coefficient) AND variation (e.g. standard deviation) or associated estimates of uncertainty (e.g. confidence intervals)
☐	☒	For null hypothesis testing, the test statistic (e.g. *F*, *t*, *r*) with confidence intervals, effect sizes, degrees of freedom and *P* value noted<br>*Give P values as exact values whenever suitable.*
☒	☐	For Bayesian analysis, information on the choice of priors and Markov chain Monte Carlo settings
☒	☐	For hierarchical and complex designs, identification of the appropriate level for tests and full reporting of outcomes
☒	☐	Estimates of effect sizes (e.g. Cohen's *d*, Pearson's *r*), indicating how they were calculated

Our web collection on statistics for biologists contains articles on many of the points above.

Software and code

Policy information about availability of computer code

Data collection	(1)All POLOS data is collected from home-built imaging system through Micro-Manager 2.0 gamma1.<br>(2)3D SIM data is collected by the commercial OMX-SIM system (Delta Vision, OMX SR, GE,USA).
Data analysis	(1) The reconstruction of POLOS image is performed with home-written Matlab (2018) codes (https://github.com/minor-planet/SPOT/) and Hilo (http://sites.bu.edu/biomicroscopy/resources/)<br>(2) Quantitative measurement is realized on Fiji based on ImageJ v1.53c<br>(3) All statistical analysis is performed in GraphPad Prism 7 and SPSS 26.0.

For manuscripts utilizing custom algorithms or software that are central to the research but not yet described in published literature, software must be made available to editors and reviewers. We strongly encourage code deposition in a community repository (e.g. GitHub). See the Nature Research guidelines for submitting code & software for further information.

Data

Policy information about availability of data

All manuscripts must include a data availability statement. This statement should provide the following information, where applicable:
- Accession codes, unique identifiers, or web links for publicly available datasets
- A list of figures that have associated raw data
- A description of any restrictions on data availability

The data that support the findings of this study are available through “figshare.com” with the identifier(s) “https://doi.org/10.6084/m9.figshare.13100471.v1”.

Field-specific reporting

Please select the one below that is the best fit for your research. If you are not sure, read the appropriate sections before making your selection.

- [x] Life sciences
- [ ] Behavioural & social sciences
- [ ] Ecological, evolutionary & environmental sciences

For a reference copy of the document with all sections, see nature.com/documents/nr-reporting-summary-flat.pdf

Life sciences study design

All studies must disclose on these points even when the disclosure is negative.

Sample size	We counted 24 different organelles in Fig3c-h ,so the sample size n=24.<br>In Figure 4b-e, we counted the emission ratio and modulation depth values of different pixel numbers. The specific pixel numbers have been listed in the manuscript respectively.<br>n=48 in Fig S6a, n=29 in Fig S6b and n=48 in Fig S6d.<br>These sample sizes are obtained based on references or previous experience.
Data exclusions	To avoid the influence of motion drift and photo-bleaching on the modulation depth measurement, the Fig3d,e,g,h and FigS6b dataset was acquired repetitively and measured three times. The data were excluded when the measurement fluctuation is more than 15%, which is obtained from observation experience of large quantities of data.
Replication	Fig1b, c, d, e, f; 2a; 3a, b; 4a;and supplementary figures 2a, c, d, e, f; 3a, b; 4a, b were done in independent triplicate or more.<br>For live cell imaging experiments, three or more independent transfection of GFP and Nile Red labeling were performed during non-consecutive days. For fixed cell or fluorescent beads imaging, three or more experimental procedure were performed on independently prepared samples.
Randomization	Samples are allocated randomly.
Blinding	Blinding was not relevant to this study, because our experimental subjects are cells rather than patients.

Reporting for specific materials, systems and methods

We require information from authors about some types of materials, experimental systems and methods used in many studies. Here, indicate whether each material, system or method listed is relevant to your study. If you are not sure if a list item applies to your research, read the appropriate section before selecting a response.

Materials & experimental systems

n/a	Involved in the study	
x		Antibodies
	x	Eukaryotic cell lines
x		Palaeontology and archaeology
x		Animals and other organisms
x		Human research participants
x		Clinical data
x		Dual use research of concern

Methods

n/a	Involved in the study	
x		ChIP-seq
x		Flow cytometry
x		MRI-based neuroimaging

Eukaryotic cell lines

Policy information about cell lines

Cell line source(s)	Human osteosarcoma U2-OS cell line (HTB-96) is purchased from ATCC.
Authentication	Cell lines were authenticated by the commercial vendors.
Mycoplasma contamination	We confirm that the cell line we used was tested negative for mycoplasma contamination.
Commonly misidentified lines (See ICLAC register)	We don't use any commonly misidentified cell lines here.
